# Effects of Aerobic Exercise on Blood Lipids in People with Overweight or Obesity: A Systematic Review and Meta-Analysis of Randomized Controlled Trials

**DOI:** 10.3390/life15020166

**Published:** 2025-01-24

**Authors:** Zhuying Chen, Runyu Zhou, Xiaojie Liu, Jingqi Wang, Leiyuyang Wang, Yuanyuan Lv, Laikang Yu

**Affiliations:** 1 Beijing Key Laboratory of Sports Performance and Skill Assessment, Beijing Sport University, Beijing 100084, China; zhuying20232120126@126.com (Z.C.); wangjingqi1803189@163.com (J.W.); 2 Laboratory of Sports Stress and Adaptation of General Administration of Sport, Beijing Sport University, Beijing 100084, China; 3 Department of Strength and Conditioning Assessment and Monitoring, Beijing Sport University, Beijing 100084, China; 18953366022@163.com (R.Z.); ningyang191@outlook.com (L.W.); 4 Department of Pharmacology and Toxicology, Medical College of Wisconsin, Milwaukee, WI 53226, USA; xiaojieliu@mcw.edu; 5 China Institute of Sport and Health Science, Beijing Sport University, Beijing 100084, China

**Keywords:** aerobic exercise, overweight, obesity, blood lipid, triglyceride, total cholesterol, high-density lipoprotein, low-density lipoprotein

## Abstract

This study aimed to investigate the effects of aerobic exercise (AE) on triglyceride (TG), total cholesterol (TC), high-density lipoprotein (HDL), and low-density lipoprotein (LDL) levels in people with overweight or obesity. Searches were performed in PubMed, Scopus, Cochrane, and Web of Science, covering data up to 27 October 2023. A meta-analysis was conducted to determine the standardized mean difference (SMD) and 95% confidence interval. Nineteen studies met the inclusion criteria. AE significantly improved blood lipids in people with overweight or obesity (TG: SMD = −0.54; *p* < 0.00001; TC: SMD = −0.24; *p* = 0.003; HDL: SMD = 0.33; *p* = 0.003; LDL: SMD = −0.42; *p* = 0.0005). Both moderate-intensity and vigorous-intensity AE demonstrated significant impacts in reducing TC, TG, and LDL, whereas only moderate-intensity exercise significantly elevated HDL. Additionally, AE significantly optimized blood lipids in those with overweight, with TG being the only parameter showing improvement in individuals with obesity. Moreover, continuous AE notably improved HDL and TG, while interval AE significantly reduced TG, TC, and LDL. Lastly, a clear positive correlation emerged between the duration of the intervention and the decrease in LDL, and a distinct negative correlation was observed between session duration and the reduction of LDL.

## 1. Introduction

The total number of children, adolescents, and adults people with overweight or obesity worldwide has exceeded 2.5 billion [1], posing a significant public health challenge on a global scale [2,3]. Obesity is a chronic, relapsing, progressive disease characterized by chronic systemic inflammation and metabolic inflexibility [4,5]. Elevated systolic blood pressure, low-density lipoprotein cholesterol (LDL), fasting glucose, and percentage body fat in individuals with obesity are major risk factors for type 2 diabetes mellitus, metabolic syndrome, and chronic cardiovascular diseases (CVDs), among others [6,7]. Overweight or obesity status increases the risk of CVDs [8,9,10,11], while dyslipidemia is an important link between obesity and the development of CVDs [12]. Obesity can lead to dyslipidemia [13], including elevated total cholesterol (TC), LDL, triglycerides (TG), and reduced high-density lipoprotein (HDL) [14], which in turn accelerates atherosclerosis and sclerotic plaque formation [15], and then elevates the risk of CVDs. Therefore, obesity is one of the major modifiable risk factors for non-communicable diseases. Recent studies emphasize the significance of management and prevention to mitigate disease risk among patients with obesity [1]. When dyslipidemia is present, it becomes a primary target for intervention therapy in populations with overweight or obesity. There is an imperative to discover strategies that enhance lipid metabolism in individuals with overweight or obesity. Thus, timely improvement and prevention measures are particularly crucial for individuals with overweight or obesity.

Currently, the main treatment modalities for obesity include bariatric surgery, pharmacological interventions, and lifestyle interventions [16]. Exercise is an effective non-pharmacological method for treating and preventing overweight or obesity, as it increases energy expenditure and regulates body weight, thereby improving related metabolic disorders [17]. Aerobic exercise (AE), as a crucial form of physical activity, is widely acknowledged for its significant impact on enhancing metabolic health and mitigating the risk of CVDs [18,19]. AE is the best exercise for weight loss, as it improves blood lipids and effectively reduces CVD risk factors in individuals with overweight or obesity [20,21]. Regular AE can reduce the waist circumference and associated visceral adipose tissue in individuals with overweight or obesity, thereby improving lipid metabolism [22,23,24]. A four-week aerobic cycling exercise intervention effectively reduced visceral fat, TG, and plasma-free fatty acids (FFA) in men and women with obesity [25]. Another study showed that a twenty-week AE intervention effectively lowered TG and LDL and raised HDL in adolescents with obesity [26]. However, the effects and mechanisms of AE on lipids improvement are unclear [27].

Previous meta-analyses have reported that AE significantly improved TG but not other lipid profiles in people with obesity [28]. In addition, a meta-analysis showed that 6–12 months of moderate-intensity AE improved body weight and waist circumference in people with overweight or obesity, with a modest but no more benefit on blood lipids [29]. Furthermore, the research suggests that high-intensity AE may have a better intervention effect on improving visceral adipose tissue in individuals with overweight and obesity [30]. However, it has also been shown that high-intensity interval exercise does not affect insulin, lipid profile, C-reactive protein, and interleukin-6 in people with overweight or obesity [31]. To further validate the impact of AE on lipid metabolism among individuals with overweight or obesity, several randomized controlled trials (RCTs) have been conducted to evaluate the effects of AE on blood lipids [32,33,34,35]. The results of these studies demonstrate that different AE interventions have varying effects on the lipid profile. Moreover, a recent study suggested that AE primarily increases HDL levels, while reductions in TC, TG, and LDL are attributed to weight loss [36].

Numerous studies have indicated a positive impact of AE on blood lipid profiles. However, due to a lack of systematic comprehensive evaluation, there are still inconsistent conclusions regarding the effects of AE on blood lipids in individuals with overweight or obesity. Moreover, the optimal form of exercise intervention, such as moderate- or high-intensity continuous AE, or high-intensity interval training (HIIT), has yet to be definitively determined. Therefore, we conducted a comprehensive systematic review and meta-analysis to delve deeper into the modalities, intervention types, target populations, and other characteristics of AE in order to validate its impact on blood lipid profiles in individuals with overweight or obesity.

## 2. Materials and Methods

### 2.1. Design

This systematic review was conducted following the Preferred Reporting Items for Systematic Evaluation and Meta-Analyses guidelines (PRISMA, 2020) [37]. The protocol is registered with PROSPERO (CRD42024500119).

### 2.2. Search Strategy

All studies were searched in five databases: PubMed, Web of Science, Cochrane, Embase, and Scopus. We searched for studies investigating the effects of AE on blood lipid indices in individuals with overweight or obesity until 27 October 2023, using the following keywords and MESH terms: exercise, overweight, obesity, and lipids.

### 2.3. Eligibility Criteria

The eligible studies were selected independently by two authors (ZC and RZ). Disagreements were resolved by a third author (YL). The inclusion criteria for eligible studies were as follows: (1) RCTs; (2) including an AE group and a control group (no exercise intervention); (3) focusing on individuals with overweight or obesity free of other medical conditions; (4) containing measurements of at least one lipid index. Literature in languages other than English or using animal models was excluded, and reviews and conference articles were also excluded from the analysis.

### 2.4. Data Extraction

Two authors (ZC and RZ) extracted the data using a specific sheet, and any disagreements were resolved through discussion with a third author (YL). When information was unclear or disputed, the authors resolved it through consultation or by contacting the authors of the article. The following information was extracted: (1) study design, quality, and sample size; (2) subject characteristics [age, gender, health status, body mass index (BMI), and baseline lipids]; (3) intervention characteristics (frequency, intensity, and duration); (4) outcome indicators (TG, TC, HDL, and LDL).

### 2.5. Methodological Quality Assessment

The assessment of the risk of bias was carried out independently by two authors (ZC and RZ), and any discrepancies were resolved through discussion. The Cochrane Collaboration bias tool was used to assess the quality of eligible studies, covering domains such as selection bias, implementation bias, detection bias, attrition bias, and reporting bias [38]. Each domain was classified as having a “low risk”, “high risk”, or “unclear” risk of bias [39].

### 2.6. Statistical Analysis

This study employed a random-effects model to combine data, taking into account population characteristics, study design, and the heterogeneity between the studies. Effect sizes were estimated using standardized mean difference (SMD) with 95% confidence interval (CI). The outcome of the meta-analysis was the net difference in lipids between the intervention and control groups. The *I*^2^ statistic was also used to assess the heterogeneity, where < 25% indicated a low risk of heterogeneity, 25–75% represented a moderate risk of heterogeneity, and > 75% signified a high risk of heterogeneity [40,41]. In the case of heterogeneity (*I*^2^ > 50%), subgroup analysis was used to interpret the results [42].

In subgroup analyses, we attempted to categorize the included studies based on intensity (moderate-intensity, vigorous-intensity), intervention type (interval AE, continuous AE), participants’ age (adolescents, age < 18; young adults, 18 ≤ age < 45; middle-aged, 45 ≤ age < 60), and participants’ basal BMI (overweight, 25 ≤ BMI < 30; obesity, BMI ≥ 30). If the studies reported a range of intensities over time, the maximum exercise intensity at the end of the intervention was selected. Relative [maximal oxygen uptake (VO_2_max) or maximal heart rate (HRmax)] and absolute [metabolic equivalents (METs)] exercise intensities were calculated using the methods and equations described by Howley et al. [43]. In addition, the study also calculated the average weekly energy expended per week for the weekly AE intervention. Meta-regression analysis was used to test for possible a dose-response relationship between exercise characteristics (intensity, frequency, and duration) and the effect on lipid improvement. Publication bias was assessed by visual inspection of funnel plots and Egger’s test [44]. Statistical analysis was performed using RevMan 5.4 and Stata 17 (Stata Corp. 2021. College Station, TX, USA) software, with significance set at *p* < 0.05.

## 3. Results

### 3.1. Studies Selection

**Figure 1:**
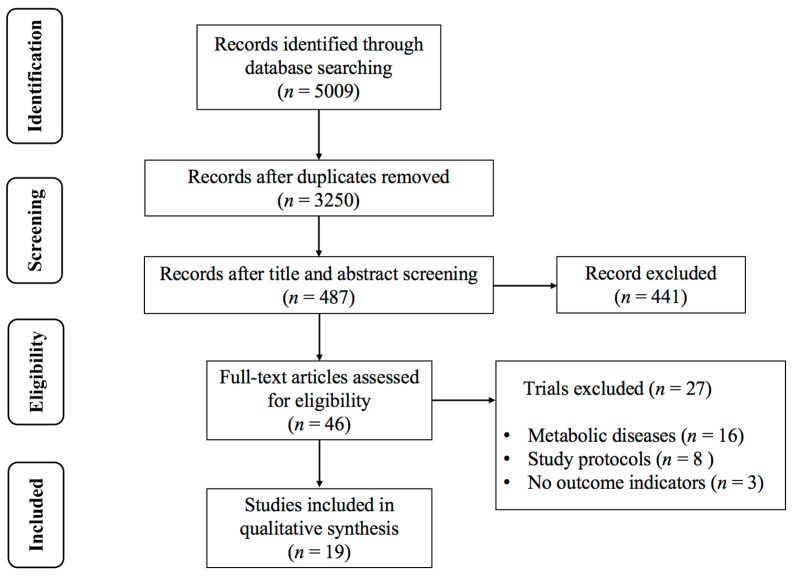
PRISMA flowchart of study selection.

The process of screening the eligible studies is summarized in Figure 1. The initial search yielded 5009 search records, of which 521 were from PubMed, 954 from Web of Science, 2248 from Cochrane, 440 from Embase, and 846 from Scopus. After excluding duplicates, 3250 studies remained. Among these, 3204 studies were deemed ineligible for inclusion based on title and abstract screening. Twenty-seven studies were excluded after reading the full text of 46 studies due to the following reasons: (1) the presence of other metabolic disorders (*n* = 8); (2) multiple exercise combined interventions (*n* = 16); and (3) inability to extracted data (*n* = 3). Finally, 19 RCTs [21,22,32,33,34,35,45,46,47,48,49,50,51,52,53,54,55,56,57] examining the effects of AE on blood lipids in individuals with overweight or obesity were considered eligible for the final analysis.

### 3.2. Characteristics of the Included Studies

The characteristics of the included studies are summarized in Table S1. The total sample size was 646, with 380 participants in the intervention groups and 266 in the control groups. The average age ranged from 11 to 75 years. Of these, six studies [21,32,33,47,48,49] involved adolescents, eight studies [22,45,50,51,52,53,54,55] involved young adults, three studies [34,35,46] involved middle-aged people, one study [56] involved old-aged, and one study [53] between young adults and middle-age. Twelve studies [21,32,34,35,46,47,48,50,52,54,56,57] involved participants with overweight and seven studies [22,32,45,47,49,51,53] involved participants with obesity. Eleven studies [21,32,33,47,48,50,52,53,54,55,57] included only males, while seven studies [34,35,45,46,49,51,56] included only females. Thirteen studies [21,22,33,34,35,46,47,48,50,52,53,56,57] examined continuous AE, while nine studies [21,32,34,45,47,49,51,54,55] examined intermittent AE. Three studies [21,34,47] included both continuous and intermittent exercises. Eleven studies [21,32,34,46,47,49,50,53,54,56,57] intervened for 12 weeks, two studies [35,45] intervened for 10 weeks, five studies [22,33,51,52,55] intervened for 8 weeks, and one study [48] intervened for 6weeks. Ten studies [21,22,33,34,45,49,52,55,56,57] controlled for diet, two studies [35,51] did not control for diet, and seven studies [32,46,47,48,50,53,54] did not mention whether diet was controlled or not.

Following the position statement on physical activity and training intensity [58], we refined the classification of AE intensity based on the specific research context. Low intensity was defined as 1.6 < METs < 3, 20% < VO_2_max < 40%, 40% < HRmax < 55%, or 8 < rating of perceived exertion (RPE) < 10. Moderate intensity was characterized by 3 < METs < 6, 40% < VO_2_max < 60%, 55% < HRmax < 70%, or 11 < RPE < 13. Vigorous intensity was delineated by 6 < METs < 9, 60% < VO_2_max < 85%, 70% < HRmax < 90%, or 14 < RPE < 16. Ten studies [21,22,33,35,46,47,49,50,51,56] conducted moderate-intensity AE and 11 studies [21,22,32,34,45,47,49,51,54,55,57] conducted vigorous-intensity AE. Five studies [21,22,47,49,51] both involved moderate and vigorous exercise. In addition, the intensity of exercise in three studies [48,52,53] ranged between moderate and vigorous.

### 3.3. Meta-Analysis

#### 3.3.1. Effects of AE on TG in People with Overweight or Obesity

**Figure 2:**
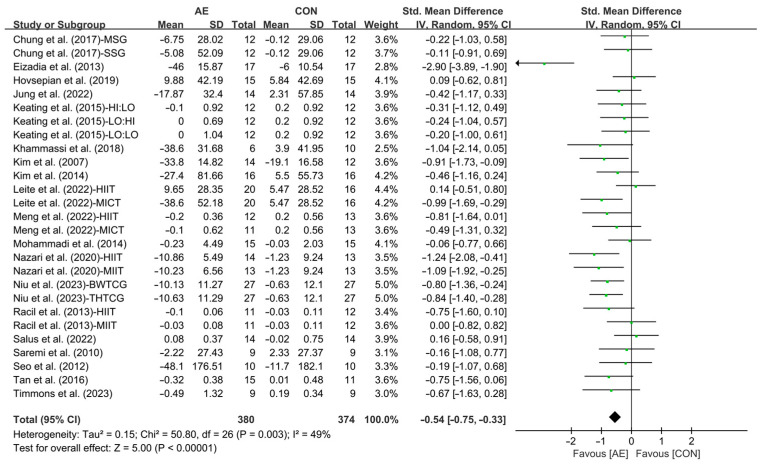
Meta-analysis results of the effects of AE on TG in people with overweight or obesity [21,22,32,33,34,35,45,46,47,48,49,50,51,52,53,54,55,56,57].

Due to the diverse units of measurement used in the studies included in the analysis, SMD was employed for comparison. All studies investigated the effect of AE on TG levels and our results showed that AE significantly reduced the TG levels in people with overweight or obesity (SMD = −0.54; 95% CI: −0.75 to −0.33, *p* < 0.00001, *I*^2^ = 49%, Figure 2).

**Figure 3:**
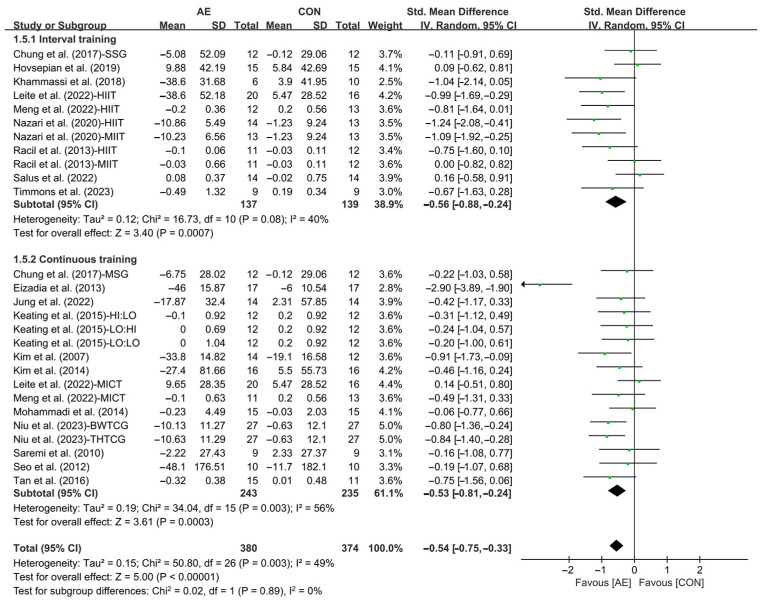
Meta-analysis results of the effects of interval and continuous AE on TG in people with overweight or obesity [21,22,32,33,34,35,45,46,47,48,49,50,51,52,53,54,55,56,57].

Subgroup analysis showed that interval AE (SMD = −0.56; 95% CI: −0.88 to −0.24, *p* = 0.0007, *I*^2^ = 40%) and continuous AE (SMD = −0.53; 95% CI: −0.81 to −0.24, *p* = 0.0003, *I*^2^ = 56%, Figure 3 and Table S2) significantly reduced the TG levels in people with overweight or obesity, with interval AE having a greater effect.

**Figure 4:**
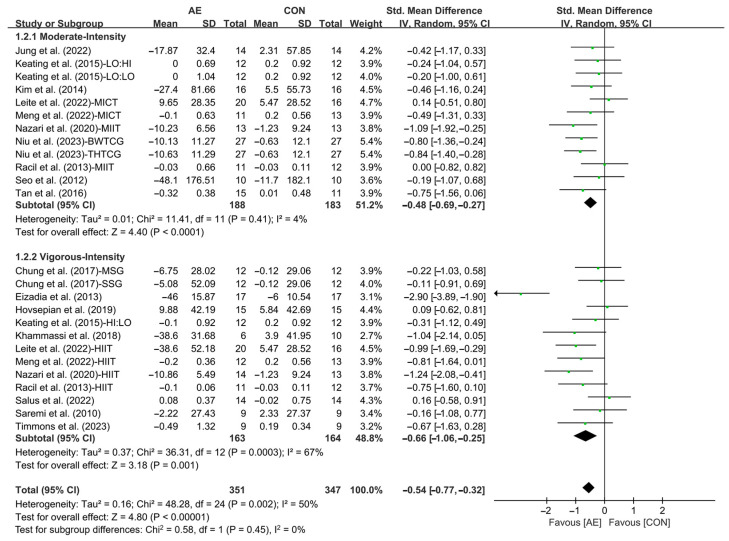
Meta-analysis results of the effects of moderate- and vigorous-intensity AE on TG in people with overweight or obesity [21,22,32,33,34,35,45,46,47,49,50,51,53,54,55,56,57].

In addition, when analyzing the subgroup by intensity, moderate-intensity AE (SMD = −0.48; 95% CI: −0.69 to −0.27, *p* < 0.0001, *I*^2^ = 4%) and vigorous-intensity AE (SMD = −0.66; 95% CI: −1.06 to −0.25, *p* = 0.001, *I*^2^ = 67%, Figure 4 and Table S2) significantly reduced the TG levels in people with overweight or obesity, with vigorous-intensity AE having a greater effect.

**Figure 5:**
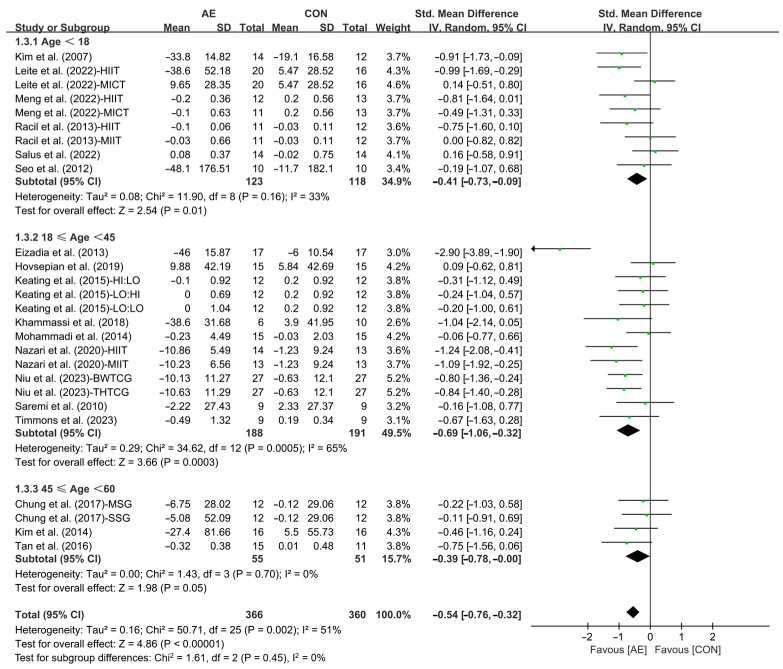
Meta-analysis results of the effects of AE on TG in people with overweight or obesity of different ages [21,22,32,33,34,35,45,46,47,48,49,50,51,52,53,54,55,57].

Furthermore, when analyzing the subgroup by participants’ age, AE significantly reduced the TG levels in adolescents (SMD = −0.41; 95% CI: −0.73 to −0.09, *p* = 0.01, *I*^2^ = 33%) and young adults with overweight or obesity (SMD = −0.69; 95% CI: −1.06 to −0.32, *p* = 0.0003, *I*^2^ = 65%), with a greater effect observed in young adults. However, AE had no significant effect on improving TG levels in middle-aged people with overweight or obesity (SMD = −0.39; 95% CI: −0.78 to −0.00, *p* = 0.05, *I*^2^ = 0%, Figure 5 and Table S2).

**Figure 6:**
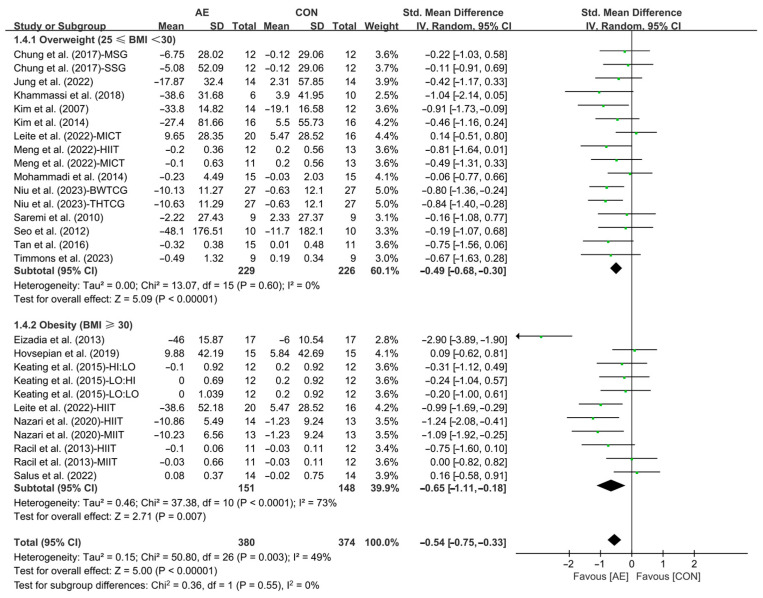
Meta-analysis results of the effects of AE on TG in people with overweight or obesity of different basal BMI [21,22,32,33,34,35,45,46,47,48,49,50,51,52,53,54,55,56,57].

Finally, when analyzing the subgroup by participants’ basal BMI, AE significantly reduced the TG levels in people with overweight (SMD = −0.49; 95% CI: −0.68 to −0.30, *p* < 0.00001, *I*^2^ = 0%) and people with obesity (SMD = −0.65; 95% CI: −1.11 to −0.18, *p* = 0.007, *I*^2^ = 73%, Figure 6 and Table S2), with a greater effect observed in people with obesity.

#### 3.3.2. Effects of AE on TC in People with Overweight or Obesity

**Figure 7:**
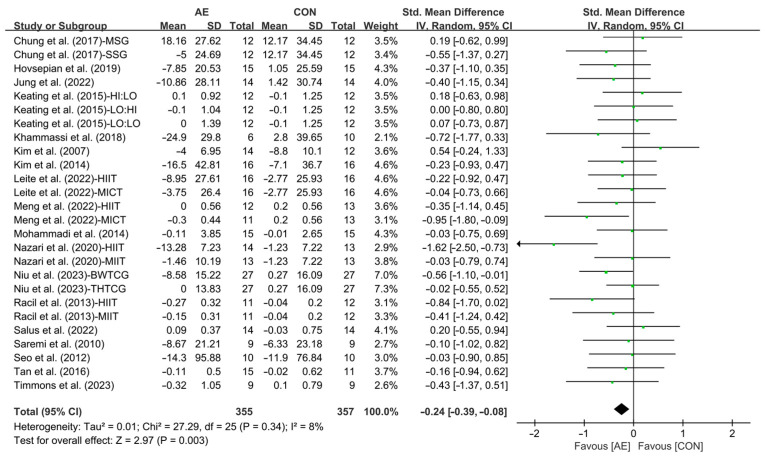
Meta-analysis results of the effects of AE on TC in people with overweight or obesity [21,22,32,33,34,35,45,46,47,48,49,50,51,52,54,55,56,57].

Our results showed that AE significantly reduced the TC levels in people with overweight or obesity (SMD = −0.24; 95% CI: −0.39 to −0.08, *p* = 0.003, *I*^2^ = 8%, Figure 7).

**Figure 8:**
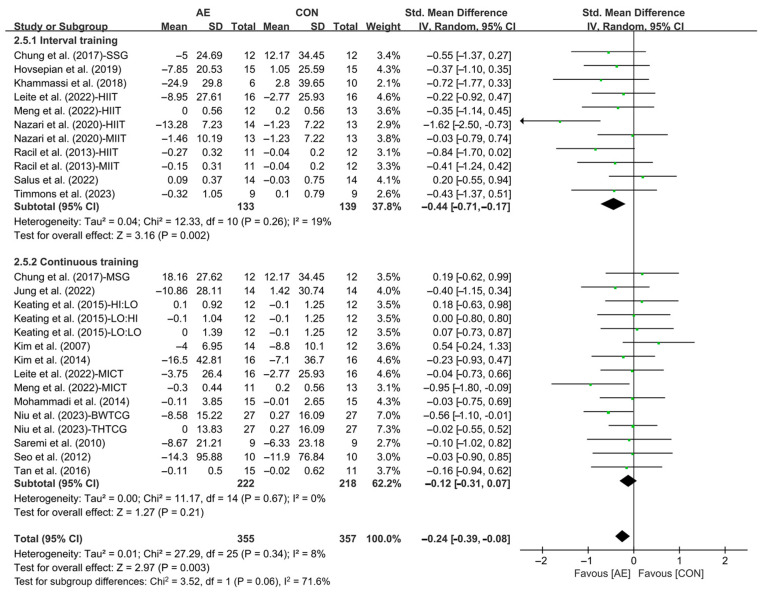
Meta-analysis results of the effects of interval and continuous AE on TC in people with overweight or obesity [21,22,32,33,34,35,45,46,47,48,49,50,51,52,54,55,56,57].

Subgroup analysis showed that interval AE (SMD = −0.44; 95% CI: −0.71 to −0.17, *p* = 0.002, *I*^2^ = 19%) significantly reduced the TC levels in people with overweight or obesity. However, continuous AE (SMD = −0.12; 95% CI: −0.31 to 0.07, *p* = 0.21, *I*^2^ = 0%, Figure 8 and Table S2) had no significant effect on improving TC in people with overweight or obesity.

**Figure 9:**
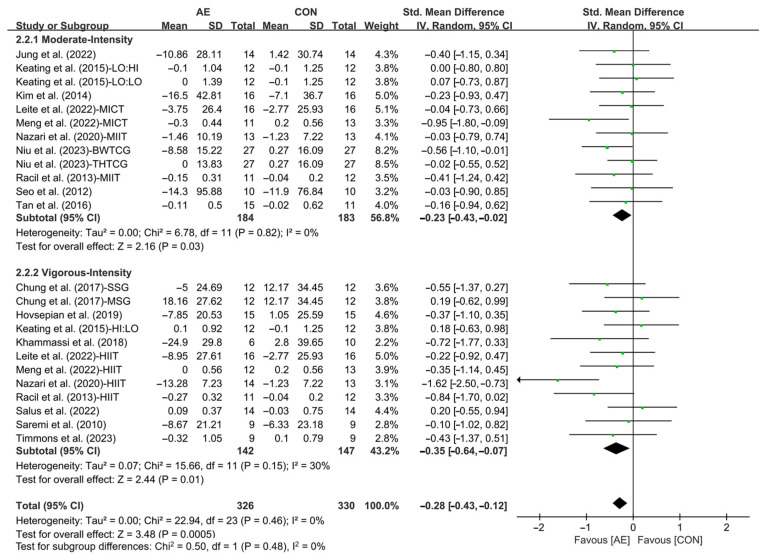
Meta-analysis results of the effects of moderate- and vigorous-intensity AE on TC in people with overweight or obesity [21,22,32,33,34,35,45,46,47,49,50,51,54,55,56,57].

In addition, when analyzing the subgroup by intensity, moderate-intensity AE (SMD = −0.23; 95% CI: −0.43 to −0.02, *p* = 0.03, *I*^2^ = 0%) and vigorous-intensity AE (SMD = −0.35; 95% CI: −0.64 to −0.07, *p* = 0.01, *I*^2^ = 30%, Figure 9 and Table S2) significantly reduced the TC levels in people with overweight or obesity, with vigorous-intensity AE having a greater effect.

**Figure 10:**
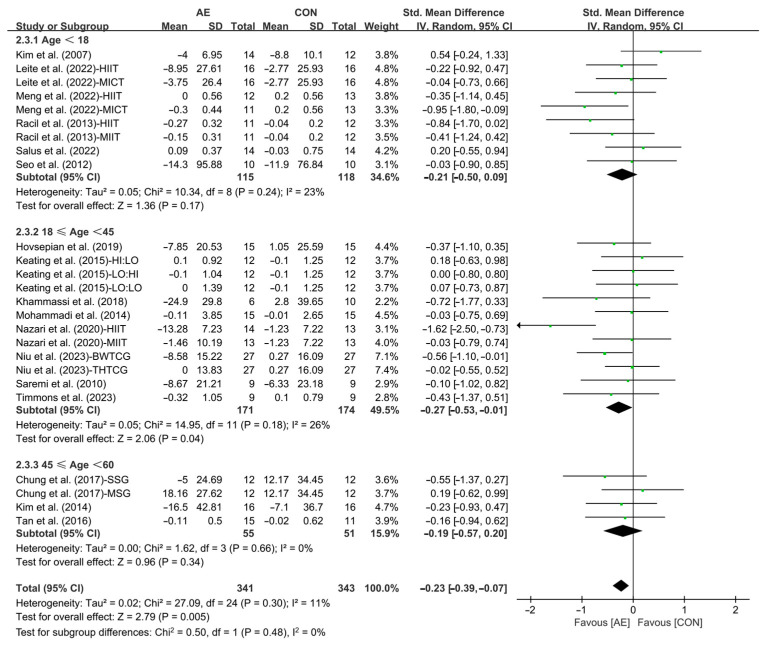
Meta-analysis results of the effects of AE on TC in people with overweight or obesity of different ages [21,22,32,33,34,35,45,46,47,48,49,50,51,52,54,55,57].

Furthermore, when analyzing the subgroup by participants’ age, AE significantly reduced the TC levels in young adults with overweight or obesity (SMD = −0.27; 95% CI: −0.53 to −0.01, *p* = 0.04, *I*^2^ = 26%). However, AE had no significant effect on improving TC levels in adolescents (SMD = −0.21; 95% CI: −0.50 to −0.09, *p* = 0.17, *I*^2^ = 23%) and middle-aged people with overweight or obesity (SMD = −0.19; 95% CI: −0.57 to −0.20, *p* = 0.34, *I*^2^ = 0%, Figure 10 and Table S2).

**Figure 11:**
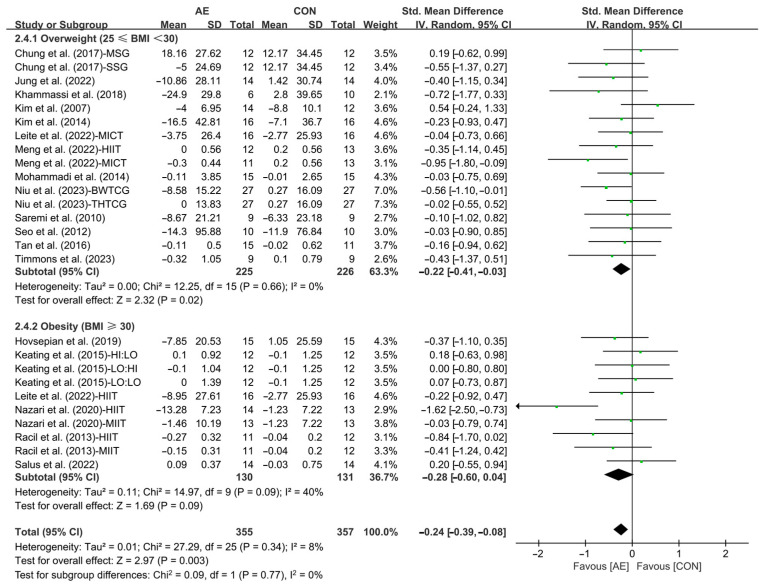
Meta-analysis results of the effects of AE on TC in people with overweight or obesity of different basal BMI [21,22,32,33,34,35,45,46,47,48,49,50,51,52,54,55,56,57].

Finally, when analyzing the subgroup by participants’ basal BMI, AE significantly reduced the TC levels in people with overweight (SMD = −0.22; 95% CI: −0.41 to −0.03, *p* = 0.02, *I*^2^ = 0%). However, AE had no significant effect on improving TC levels in people with obesity (SMD = −0.28; 95% CI: −0.60 to −0.04, *p* = 0.09, *I*^2^ = 40%, Figure 11 and Table S2).

#### 3.3.3. Effects of AE on HDL in People with Overweight or Obesity

**Figure 12:**
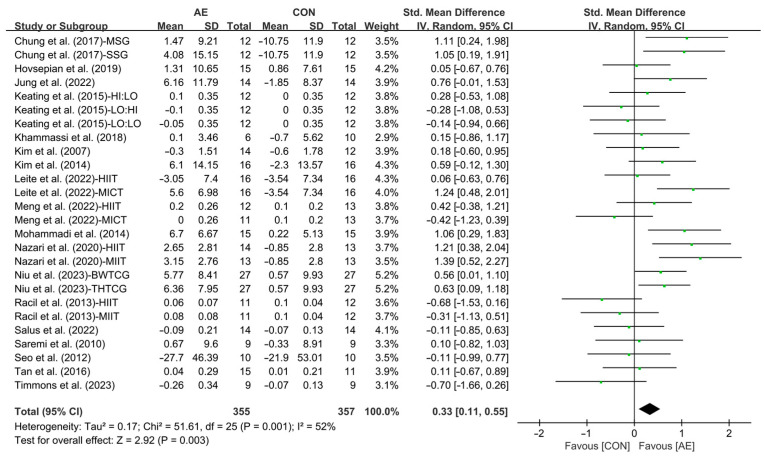
Meta-analysis results of the effects of AE on HDL in people with overweight or obesity [21,22,32,33,34,35,45,46,47,48,49,50,51,52,54,55,56,57].

Our results showed that AE significantly increased the HDL levels in people with overweight or obesity (SMD = 0.33; 95% CI: 0.11 to 0.55, *p* = 0.003, *I*^2^ = 52%, Figure 12).

**Figure 13:**
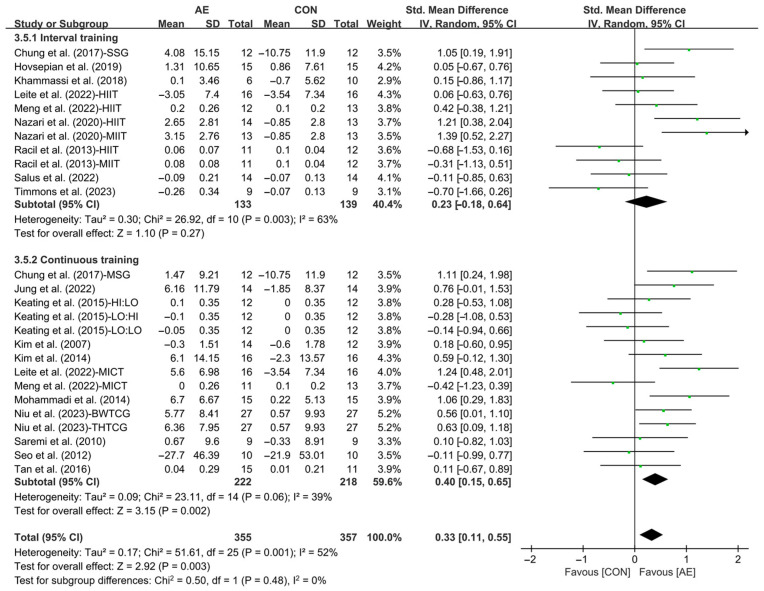
Meta-analysis results of the effects of interval and continuous AE on HDL in people with overweight or obesity [21,22,32,33,34,35,45,46,47,48,49,50,51,52,54,55,56,57].

Subgroup analysis showed that continuous AE had a significant effect on improving HDL levels in people with overweight or obesity (SMD = 0.40; 95% CI: 0.15 to 0.65, *p* = 0.002, *I*^2^ = 39%, Figure 13 and Table S2). However, interval AE (SMD = 0.23; 95% CI: −0.18 to 0.64, *p* = 0.27, *I*^2^ = 63%) had no significant effect on HDL levels in people with overweight or obesity.

**Figure 14:**
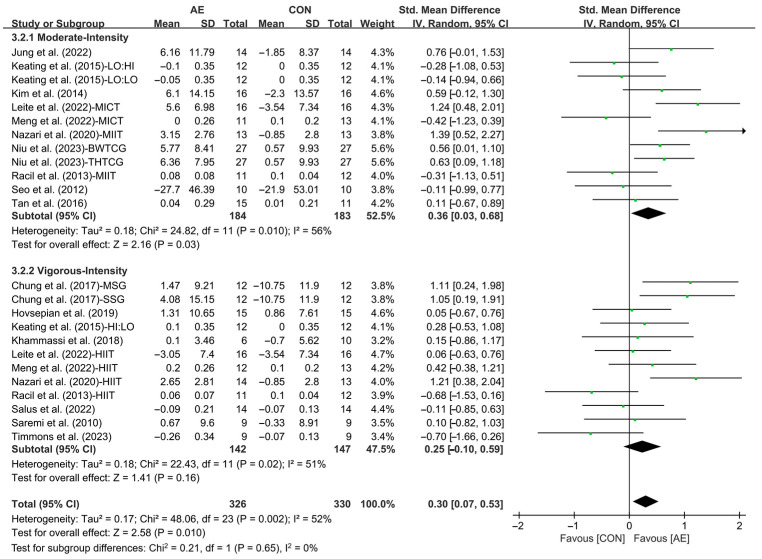
Meta-analysis results of the effects of moderate- and vigorous-intensity AE on HDL in people with overweight or obesity [21,22,32,33,34,35,45,46,47,49,50,51,54,55,56,57].

In addition, when analyzing the subgroup by intensity, moderate-intensity AE (SMD =0.36; 95% CI: 0.03 to 0.68, *p* = 0.03, *I*^2^ = 56%) significantly improved the HDL levels in people with overweight or obesity. However, vigorous-intensity AE had no significant effect on improving HDL levels in people with overweight or obesity (SMD = 0.25; 95% CI: −0.10 to 0.59, *p* = 0.16, *I*^2^ = 51%, Figure 14 and Table S2).

**Figure 15:**
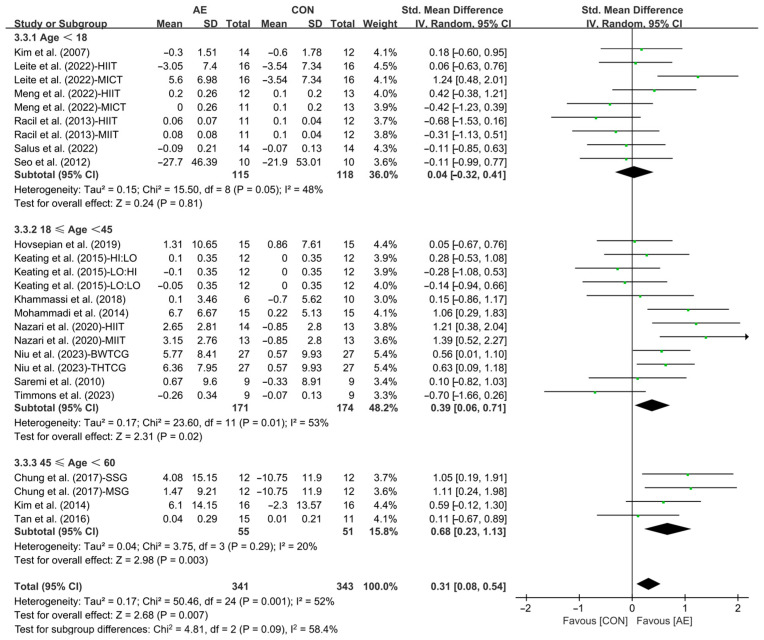
Meta-analysis results of the effects of AE on HDL in people with overweight or obesity of different ages [21,22,32,33,34,35,45,46,47,48,49,50,51,52,54,55,57].

Furthermore, when analyzing the subgroup by participants’ age, AE significantly elevated the HDL levels in young adults (SMD = 0.39; 95% CI: 0.06 to 0.71, *p* = 0.02, *I*^2^ = 53%) and middle-aged people with overweight or obesity (SMD = 0.68; 95% CI: 0.23 to 1.13, *p* = 0.003, *I*^2^ = 20%), with a greater effect observed in middle-aged people. However, AE had no significant effect on improving HDL levels in adolescents with overweight or obesity (SMD = 0.04; 95% CI: −0.32 to 0.41, *p* = 0.81, *I*^2^ = 48%, Figure 15 and Table S2).

**Figure 16:**
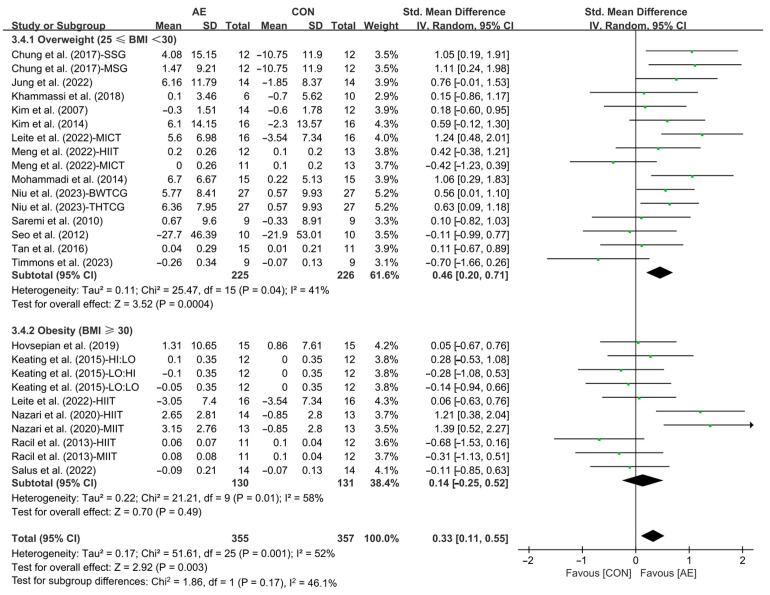
Meta-analysis results of the effects of AE on HDL in people with overweight or obesity of different basal BMI [21,22,32,33,34,35,45,46,47,48,49,50,51,52,54,55,56,57].

Finally, when analyzing the subgroup by participants’ basal BMI, AE significantly increased the HDL levels in people with overweight (SMD = 0.46; 95% CI: 0.20 to 0.71, *p* = 0.0004, *I*^2^ = 41%). However, AE had no significant effect on improving HDL levels in people with obesity (SMD = 0.14; 95% CI: −0.25 to 0.52, *p* = 0.49, *I*^2^ = 58%, Figure 16 and Table S2).

#### 3.3.4. Effects of AE on LDL in People with Overweight or Obesity

**Figure 17:**
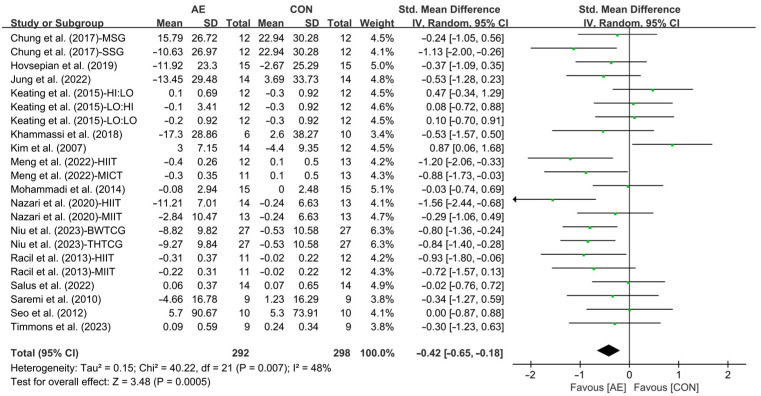
Meta-analysis results of the effects of AE on LDL in people with overweight or obesity [21,22,32,33,34,45,48,49,50,51,52,54,55,56,57].

Our results showed that AE significantly reduced the LDL levels in people with overweight or obesity (SMD = −0.42; 95% CI: −0.65 to −0.18, *p* =0.0005, *I*^2^ = 48%, Figure 17).

**Figure 18:**
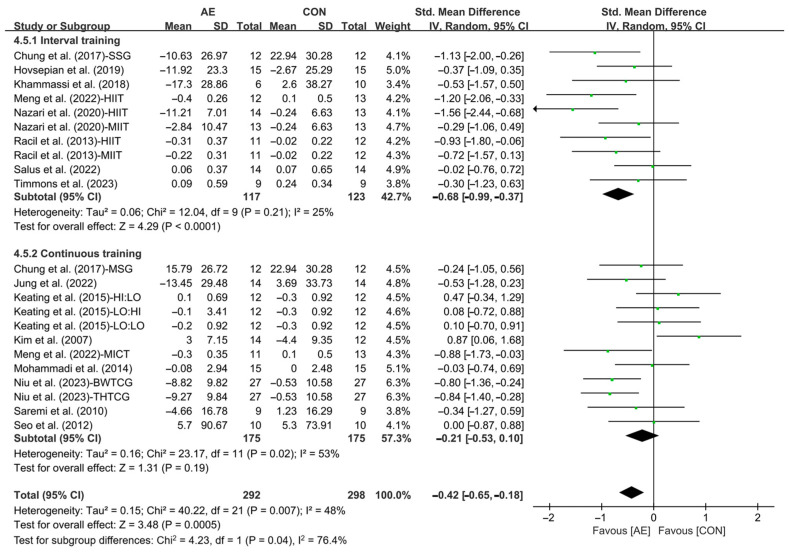
Meta-analysis results of the effects of interval and continuous AE on LDL in people with overweight or obesity [21,22,32,33,34,45,48,49,50,51,52,54,55,56,57].

Subgroup analysis showed that interval AE (SMD = −0.68; 95% CI: −0.99 to −0.37, *p* < 0.0001, *I*^2^ = 25%) significantly reduced the LDL levels in people with overweight or obesity. However, continuous AE had no significant effect on LDL in people with overweight or obesity (SMD = −0.21; 95% CI: −0.53 to 0.10, *p* = 0.19, *I*^2^ = 53%, Figure 18 and Table S2).

**Figure 19:**
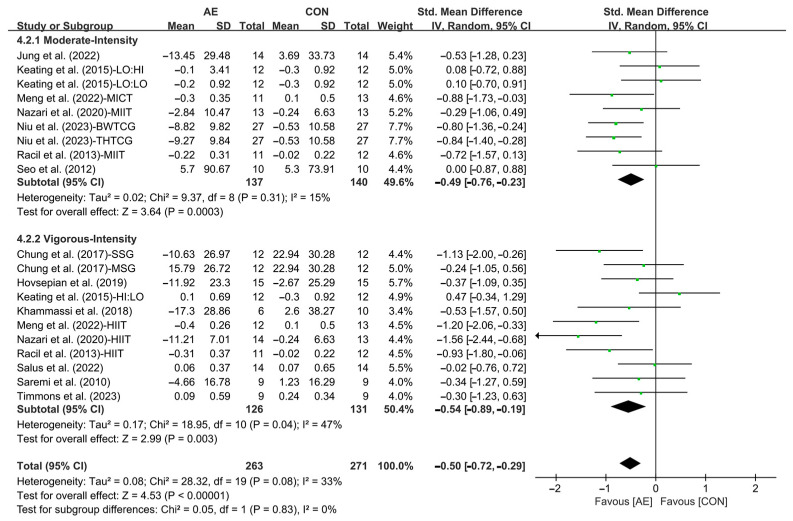
Meta-analysis results of the effects of moderate- and vigorous-intensity AE on LDL in people with overweight or obesity [21,22,32,33,34,45,49,50,51,54,55,56,57].

In addition, when analyzing the subgroup by intensity, moderate-intensity AE (SMD = −0.49; 95% CI: −0.76 to −0.23, *p* = 0.0003, *I*^2^ = 15%) and vigorous-intensity AE (SMD = −0.54; 95% CI: −0.89 to −0.19, *p* = 0.003, *I*^2^ = 47%, Figure 19 and Table S2) significantly reduced the LDL levels in people with overweight or obesity, with vigorous-intensity AE having a greater effect.

**Figure 20:**
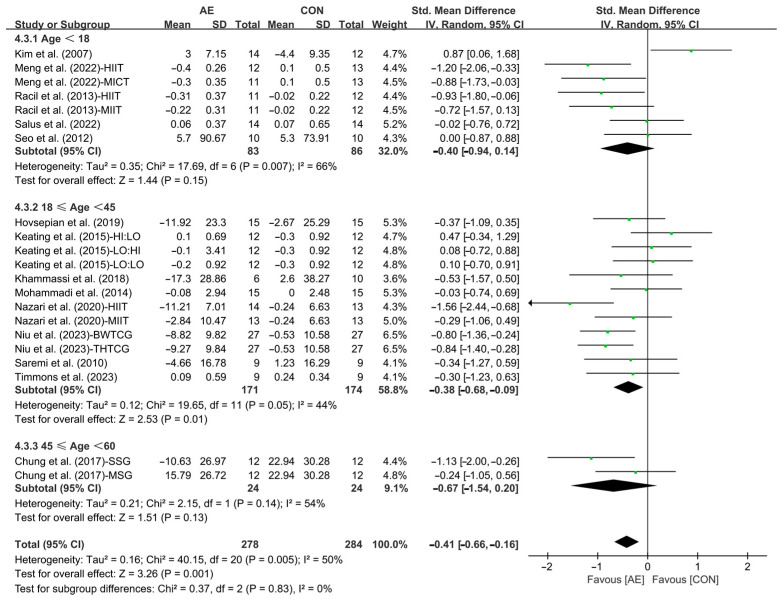
Meta-analysis results of the effects of AE on LDL in people with overweight or obesity of different ages [21,22,32,33,34,45,48,49,50,51,52,54,55,57].

Furthermore, when analyzing the subgroup by participants’ age, AE significantly reduced the LDL levels in young adults with overweight or obesity (SMD = −0.38; 95% CI: −0.68 to −0.09, *p* = 0.01 *I*^2^ = 44%). However, AE had no significant effect on improving LDL levels in adolescents (SMD = −0.40; 95% CI: −0.94 to 0.14, *p* = 0.15, *I*^2^ = 66%) and middle-aged people with overweight or obesity (SMD = −0.67; 95% CI: −1.54 to 0.20, *p* = 0.13, *I*^2^ = 54%, Figure 20 and Table S2).

**Figure 21:**
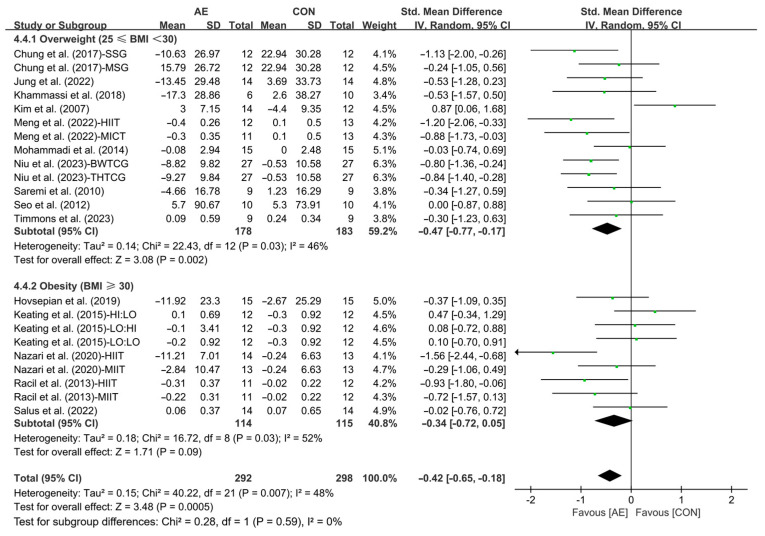
Meta-analysis results of the effects of AE on LDL in people with overweight or obesity of different basal BMI [21,22,32,33,34,45,48,49,50,51,52,54,55,56,57].

Finally, when analyzing the subgroup by participants’ basal BMI, AE significantly reduced the LDL levels with a greater effect observed in people with overweight (SMD = −0.47; 95% CI: −0.77 to −0.17, *p* = 0.002, *I*^2^ = 46%), while people with obesity (SMD = −0.34; 95% CI: −0.72 to 0.05, *p* = 0.09, *I*^2^ = 52%, Figure 21 and Table S2) had no significant effect on improving LDL levels.

### 3.4. Meta-Regression Analyses

Meta-regression analyses were performed on weekly deficits, intervention duration, session duration, frequency, relative intensity (VO_2_max/HRmax), and absolute intensity (METs). There were no significant associations between weekly deficits, intervention duration, session duration, frequency, relative intensity, or absolute intensity, and TG, TC, or HDL levels (Table S3). However, there were significant associations between session duration (*p* = 0.034), intervention duration (*p* = 0.002), and LDL levels (Figure S1). The more negative the effect size, the more effective the intervention was at lowering LDL levels. Notably, with the prolonged session duration, there was a smaller reduction in LDL levels. However, as the intervention duration increased, a more significant improvement in LDL levels was observed.

### 3.5. Risk of Bias

The Cochrane risk assessment tool was utilized to evaluate the methodological quality of the included studies across six domains: selection bias, performance bias, detection bias, attrition bias, reporting bias, and other biases. The quality assessment was categorized into three levels: low risk, high risk, and unclear risk. The quality assessment results revealed that five studies exhibited a low risk of bias, five studies exhibited a high risk of bias, and the others exhibited an unclear risk of bias (Figure S2). Publication bias was visually evaluated through funnel plot inspection to ensure a comprehensive assessment of the included studies (Figure S3). The results of the Egger’s test indicated that the small sample size studies were not enough to affect the final results (TG, *p* = 0.361; TC, *p* = 0.322; HDL, *p* = 0.719; and LDL, *p* = 0.637, Table S4).

### 3.6. Sensitivity Analyses

Sensitivity analyses revealed that the overall effect of AE on TG (Figure S4), TC (Figure S5), HDL (Figure S6), and LDL (Figure S7) in people with overweight or obesity remained consistent in terms of direction and compatibility levels when any of the included studies were omitted.

## 4. Discussion

In this study, we found that AE significantly increased HDL levels and simultaneously reduced TG, TC, and LDL levels in individuals with overweight or obesity, which is consistent with a previous study, showing that a 12-week AE significantly improved TG, TC, LDL, and HDL levels, as well as reduced cardiometabolic risk factors in women with obesity [59]. Another study demonstrated that a 6-week AE in young men with obesity significantly reduced TG, TC, and LDL levels [60]. In addition, the 12-week AE significantly reduced TC, TG, and LDL levels in male college students with obesity, with higher intensity intervals having better effects [61]. The 4-week HIIT significantly improved the lipid metabolism of female students with obesity by reducing TC, TG, LDL, and TC/HDL ratios and increasing HDL levels [62]. AE can reduce the interaction between lipid droplets and mitochondria, thereby reducing fat accumulation in the liver and improving lipid metabolism [63]. Furthermore, AE increases fatty acid oxidation (FAO), which promotes fat metabolism and aids in lowering blood lipid levels [5,64,65]. Therefore, the mechanism of how exercise improves blood lipids can be summarized in the following two aspects. On the one hand, AE can increase whole-body fat oxidation, adipose tissue lipolysis, and fatty acid utilization by skeletal muscle [66]. On the other hand, AE can also regulate intracellular lipid delivery and mitochondrial β-oxidation, encouraging the muscle to absorb and utilize more FFA and cholesterol [67,68]. This may increase the reverse transport capacity of TC, resulting in a decrease in LDL and an increase in HDL in the blood. While some previous studies have explored the potential physiological mechanism of exercise for improving blood lipids, there is still no clear explanation [69,70].

Recent studies have demonstrated that obesity is a metabolic dysregulation caused by reactive oxygen species (ROS), which contribute to ribotoxic stress response (RSR) through the activation of Zinc finger protein kinase alpha (ZAKα) by participating in redox reactions [71]. Therefore, AE may positively influence lipid metabolism in individuals with obesity by enhancing cellular antioxidant capacity, reducing oxidative stress, and balancing ROS levels. This differs from the results of a previous meta-analysis study. Cai et al. [28] concluded that AE significantly reduced TG levels in adults with obesity, but had no significant effect on TC, HDL, and LDL. Another meta-analysis showed that AE lasting greater than 8 weeks only significantly reduced TG in adults with overweight or obesity [72]. Usually, exercise affects the size of TG-rich lipoproteins, enhances lipoprotein lipase activity, increases muscle uptake of TG, and decreases hepatic VLDL-TG output. Together, these mechanisms lead to a decrease in TG levels [63]. In addition, decreased TG may be associated with AE-induced fatty acid oxidation. The decrease in TG levels may be attributed to the fatty acid oxidation induced by AE. The insignificance of other indicators can be attributed to factors such as small sample size, heterogeneity, and individual influencing variables, including gender, age, and diet. Batrakoulis et al. [73] supported the notion that AE reduced TG, while still exhibiting inconsistency in its effectiveness with regard to other blood lipids. In addition, Taghian et al. [74] demonstrated that 12 weeks of moderate-intensity AE could lower TG and increase HDL in women with obesity. Furthermore, Chiu et al. [75] also supported a 12-week walking intervention that reduced TG and increased HDL in female college students with obesity. The reason for this is that AE increases the mitochondrial density of muscle, increases oxidase activity, improves the activity of fat oxidase and lipoprotein lipase, and promotes lipid metabolism, thereby reducing TG and increasing HDL. Only 12 weeks of AE at the lowest respiratory quotient (RQ) intensity could reduce TG in women with overweight, compared to ventilate threshold (VT) intensity [76]. This may imply that low-intensity AE is more conducive to fat oxidation, thus lowering TG. Previous studies have demonstrated that AE can effectively reduce TG in children, adolescents, and adults with overweight or obesity [72,77]. The discrepancies in the results may be attributed to variations in training programs, measurement techniques, gender, age group (child, adolescent, young adult, or elderly), or the level of obesity (overweight or obesity), among other factors. Therefore, different lipid indexes have different influence mechanisms, so more studies are needed to elucidate the mechanism.

The results of the subgroup analysis showed that both moderate-intensity and vigorous-intensity AE significantly reduced TG, TC, and LDL levels. Vigorous-intensity AE had a greater effect on lipids in people with overweight or obesity. In contrast, only moderate-intensity AE significantly elevated HDL levels. This is possibly due to the induction of fatty acid oxidation leading to TG level reduction [78] and the associated increase in lipoprotein affinity [79]. Consistent with the results of this study, the meta-analysis results of Li et al. [80] showed that AE improved TG, TC, and LDL in adolescents with obesity, but did not significantly improve HDL. A further meta-analysis of children with obesity also showed that vigorous-intensity exercise did not considerably improve HDL [81]. A recent meta-analysis comparing moderate-intensity and vigorous-intensity AE for lipid improvement in adolescents with obesity has also demonstrated that vigorous-intensity AE led to a significant reduction in LDL and TC levels, but did not result in a significant increase in HDL levels [82]. A randomized controlled trial conducted in adolescents with obesity revealed that a 12-week vigorous-intensity AE did not significantly improve HDL levels [54]. In addition, a recent RCT showed that 6 weeks of different-intensity AE did not have a significant effect on HDL levels in adult men with obesity, particularly when vigorous-intensity exercise was used [60]. A previous review of vigorous-intensity AE in people with obesity has shown that at least 8 weeks of intervention is required to achieve a significant increase in HDL [83]. This may be because low HDL levels are associated with overweight or obesity, and HDL levels only increase when the threshold for exercise intensity and intervention duration is reached [84].

Inconsistent with our results, one study showed that both a 12-week moderate-intensity and vigorous-intensity AE increased HDL in adult men with obesity. The results showed that vigorous-intensity AE had a greater effect on lipolysis in adipose tissue than the moderate-intensity regimen. The reason for this is that vigorous-intensity AE leads to higher metabolic stress, which increases catecholamines and hormones (such as growth hormone and glucagon) levels in an intensity-related manner [85]. Excessive exercise intensity occurs when the intensity, frequency, or duration of vigorous exercise exceeds an individual’s fitness level, leading to inadequate recovery and negatively impacting HDL levels. Therefore, moderate- and vigorous-intensity exercise may be more beneficial for increasing HDL levels, while excessive exercise may lead to a reversing effect. Previous studies have demonstrated the effects of regular AE on metabolic adaptation and lipid improvement, yet there is still no consensus on determining the optimal training intensity for improving blood lipids [5,64]. Okura et al. [86] investigated a 14-week intervention of walking (low-intensity) and AE (70−85% VO_2_max) in women with obesity and found that vigorous-intensity AE significantly reduced LDL, whereas Racil et al. [49] suggested that both moderate-intensity and vigorous-intensity interval training reduced total body fat in young women with obesity, with only vigorous-intensity exercise reducing TG and TC. Furthermore, a more pronounced hemostatic effect on Salusin-α, a protective biomarker of atherosclerosis, was observed during moderate-intensity interval training, whereas Salusin-β, a precursor of lipids, was more affected during vigorous-intensity interval training. This is the mechanism by which different intensities of exercise affect lipids [51]. Therefore, for people with overweight or obesity, the effects of moderate- and vigorous-intensity AE on TG and LDL may play a key role in reducing CVD risk.

The bigger debate revolves around the effect of different forms of AE on improving blood lipids. Our subgroup analysis showed that interval AE could lower TG, TC, and LDL levels, but did not significantly increase HDL levels, whereas continuous AE lowered TG and increased HDL levels, yet failed to lower TC and LDL levels. However, only continuous AE significantly increased HDL levels, aligning with findings suggesting that continuous training has a greater effect on HDL [47,87]. A meta-study on children with obesity showed that high-intensity interval exercise did not significantly improve HDL, whereas continuous AE did [81]. Another meta-analysis comparing moderate-intensity continuous training (MICT) and HIIT for lipid improvement in youth with obesity also indicated that HIIT significantly reduced LDL and TC levels, but did not significantly affect HDL [82]. Furthermore, continuous exercise has demonstrated limited efficacy in decreasing TC and LDL. In line with our results, an RCT of 16 weeks of interval and continuous moderate-intensity intervention in women with obesity showed no significant improvement in TC and LDL [88]. It is well-known that visceral fat is one of the main factors closely related to high TC levels, and reducing visceral fat is an effective way to lower TC levels [25,63]. The mechanism behind the ability of high-intensity intermittent AE to reduce TC levels is mainly achieved by reducing visceral fat. Studies have shown that HIIT is more effective than MICT in stimulating the secretion of fat-soluble hormones such as catecholamines [89]. Norepinephrine promotes fat oxidation, while epinephrine promotes carbohydrate oxidation and fat solubility. Fat oxidation after HIIT training may be indirectly related to increased sympathetic nervous system (SNS) activation (via norepinephrine release), ultimately leading to increased levels of circulating FFA post-exercise, regulating lipid metabolism.

Continuous AE can also improve TC, but it mainly depends on the intensity and duration of the intervention. Some studies may have only conducted short-term exercise interventions, leaving insufficient time to observe the effects of exercise on blood lipids. Sustained moderate-intensity exercise may require a longer time to demonstrate significant effects. Therefore, there may be some limitations and challenges to the effect of continuous moderate-intensity exercise on reducing TC and LDL, and more studies are needed to explore the mechanisms and influencing factors. Interval training has varying intervention effects, which may stem from differences in age or obesity, training variety, short duration, and incomplete consistency of intervals [51,54]. Sawyer et al. [90] reported that 8 weeks of HIIT had no significant effect on TC, TG, LDL, or HDL in adults with obesity. However, Kim et al. [48] demonstrated that 6 weeks of jump rope exercise improved TC, insulin sensitivity, and lipocalin levels in men with obesity, where plasma lipocalin levels associated with TC, HDL, and LDL were negatively correlated with body fat levels. Lipocalin is a hormone secreted by adipocytes that improves insulin sensitivity, regulates lipid metabolism, performs anti-inflammatory effects, and protects cardiovascular health. Therefore, AE interventions may further promote metabolic health and lower cholesterol levels by increasing lipocalin levels. In addition, Ouerghi et al. [91,92] demonstrated that 8 weeks of HIIT induced significant reductions in TC, LDL, and TG in young men with overweight or obesity. Moreover, continuous AE leads to a modest reduction in waist circumference and associated visceral adipose tissue [23,24]. Conversely, HIIT may have greater benefits for cardiorespiratory capacity than moderate-intensity exercise [23,87]. Therefore, the intensity and duration of AE are critical determinants that influence lipid improvement in people with overweight or obesity.

Our results found that basal BMI and age were factors influencing the effects of AE on lipids in people with overweight or obesity. Consistent with other studies, our results demonstrated that AE was more effective in improving lipids in people with overweight, with no significant improvement in HDL, TC, and LDL in people with obesity [54]. Different basal BMI significantly reduced TG. This indicated that the beneficial effects of AE on lipid markers may depend on the individual’s initial level of obesity. Additionally, there is evidence to support that AE improves TC, TG, and LDL primarily through weight loss [33,36,53]. Although the degree of weight loss is one of the key determinants of lipid changes, overweight individuals may experience more significant lipid improvements when they lose a relatively small amount of weight. This is due to their potentially healthier metabolic state compared to obese individuals. In contrast, obese individuals typically need to lose more weight to observe a similar magnitude of lipid improvement. Therefore, weight loss goals and their implications for metabolic health must be specifically considered when developing an exercise program.

As one of the main factors affecting lipids, age is an important influencing factor [93,94]. Firstly, AE has been shown to reduce TG in people with overweight or obesity of all ages. Eizadi et al. [53] conducted an AE intervention in men with obesity three times a week for three months and the results showed an increase in β-cell function after the intervention, accompanied by a decrease in body fat, blood glucose, and TG. Secondly, a significant effect on the improvement of HDL in young adults and middle-aged people, with a greater effect observed in middle-aged people. This indicated that AE can reverse the decline in β-cell function caused by aging or obesity, thus improving metabolic regulation and dyslipidemia [95]. Thirdly, AE has a significant effect on the improvement of LDL, TC, TG, and HDL in young adults. For young adults, the LDL-lowering effect is better than in other age groups. This has been supported in other studies. Mohammadi et al. [52] found significant differences in the effects of 8-week aerobic training on cardiometabolic health parameters in young men with obesity, with LDL decreasing. This is probably because young adulthood is a period of high metabolic activity [96]. Thus, AE has a positive effect on lipid metabolism in different people.

In addition, our study found a potentially significant dose-response relationship between AE, specifically in terms of the session duration and intervention duration, and LDL. Initially, the session duration was positively correlated with the reduction in LDL. This is consistent with the results of the subgroup analysis, where interval exercise significantly reduced LDL, whereas continuous exercise did not. This finding is consistent with a previous study [21], showing that in terms of session duration, short bursts of HIIT are more effective for LDL improvement.

Subsequently, LDL decreased as the intervention duration increased, suggesting that the longer the intervention duration, the better the effect on lipid improvement. In contrast to our results, Batacan et al. [31] showed that both short-term HIIT (< 12 weeks) and long-term HIIT (> 12 weeks) had no effect on insulin, lipids, C-reactive protein, and interleukin 6 in people with overweight or obesity, but they were effective in increasing VO_2_max and improving CVD risk factors. However, the mechanisms underlying the beneficial effects of regular exercise on lipid metabolism have not been fully elucidated. Prolonged AE may reduce the production and clearance of LDL and while increasing the catabolism of TG-rich lipoproteins, it may also increase TG production, ultimately reducing TG and LDL levels and improving lipid metabolism [64]. Karami et al. [97] proved that regular AE has a better intervention effect on people with obesity, and regular training can significantly affect the TC and HDL/LDL ratio of adolescents with obesity. These findings support emerging evidence that long-term regular high-intensity interval AE may be better for improving blood lipids and promoting cardiovascular health.

Some limitations should also be noted in this study. Firstly, subgroup analyses showed that exercise intensity, type, participants’ age, and basal BMI were the sources of heterogeneity in this meta-analysis, yet heterogeneity still existed between studies in the subgroup analyses. Secondly, the impact of diet on dyslipidemia remains significant. Although all included studies used dietary reviews or questionnaires to assess the greening of dietary intake. This helped to minimize potential confounding by dietary changes, but could not eliminate confounding by dietary intake. Thirdly, the included studies encompassed a wide range of AE forms (such as running and cycling) and diverse participant groups (varying in age, gender, and obesity level), which may influence the assessment of lipids and affect the applicability of the findings. Additionally, some studies had small sample sizes, and the variability in training types may further restrict the generalizability of the results. Furthermore, there is no consensus on the appropriate AE intensity for overweight and obese individuals, as it varies across studies and may affect the interpretation of exercise effects. In particular, the size of HDL and LDL particles was not discussed in this study, which limits a comprehensive assessment of lipid profiles. In this study, we analyzed the age factor, and future studies could consider the effect of factors such as gender, background, and history of physical exercise on levels in overweight and obese populations. In addition to AE, it is meaningful to explore the effects of daily physical activity on blood lipids in overweight or obese individuals.

## 5. Conclusions

AE markedly enhanced blood lipids in individuals with overweight or obesity. Both moderate-intensity and vigorous-intensity AE demonstrated significant impacts in reducing TC, TG, and LDL, whereas only moderate-intensity exercise significantly elevated HDL. Additionally, AE significantly optimized blood lipids in those with overweight, with TG being the only parameter showing improvement in individuals with obesity. Moreover, continuous AE notably improved HDL and TG, while interval AE significantly reduced TG, TC, and LDL. Lastly, a clear positive correlation emerged between the duration of the intervention and the decrease in LDL, and a distinct negative correlation was observed between session duration and the reduction of LDL.

## Data Availability

All data generated or analyzed during this study are included in the article/Supplementary Materials Section.

## Supplementary Materials

The following supporting information can be downloaded at: https://www.mdpi.com/article/10.3390/life15020166/s1, Figure S1: Meta-regression analyses results; Figure S2: Results of Cochrane risk of bias tool; Figure S3: Funnel plot; Figure S4: Sensitivity analyses results of TG; Figure S5: Sensitivity analyses results of TC; Figure S6: Sensitivity analyses results of HDL; Figure S7: Sensitivity analyses results of LDL; Table S1: Characteristics of studies included in this meta-analysis; Table S2: Results of subgroup analysis; Table S3: Meta-regression coefficients of the effect of significant moderators on selected outcomes; Table S4: Results of Egger’s test.
