# Supplementary material for: Effects of Aerobic Exercise on Blood Lipids in People with Overweight or Obesity: A Systematic Review and Meta-Analysis of Randomized Controlled Trials
Source: Life (Basel). 2025 Jan 24;15(2):166. doi: 10.3390/life15020166 (PMC11856645; doi:10.3390/life15020166)

Supplemental material

Effects of Aerobic Exercise on Blood Lipids in People with Overweight or Obesity: A Systematic Review and Meta-analysis of Randomized Controlled Trials

Table S1. Characteristics of studies included in this meta-analysis 2
Table S2. Results of subgroup analysis 7
Table S3. Meta-regression coefficients of the effect of significant moderators on selected outcomes 8
Table S4. Results of Egger's test 9
Figure S1. Meta-regression analyses results..10
Figure S2. Results of Cochrane risk of bias tool 11
Figure S3. Funnel plot 12
Figure S4. Sensitivity analyses results of TG...13
Figure S5. Sensitivity analyses results of TC 14
Figure S6. Sensitivity analyses results of HDL...15
Figure S7. Sensitivity analyses results of LDL 16

Table S1. Characteristics of studies included in this meta-analysis

Studies	Sample	Sex (F/M)	BMI (kg/m^2)	Age (y)	Intensity	Duration	Frequency	Session duration	Adherence	Indexes
Leite et al. 2022 (48)	MICT (*n* = 20);	0/22	28.53 ± 2.98	12.79 ± 1.56	95% HR_{max}	12 weeks	3 days/week	HIIT: 45-54 min	MICT: 91%;	TG, HDL, TC
	HIIT (*n* = 20);	0/20	30.67 ± 3.74		55-75% HR_{max}			MICT: 90 min	HIIT: 100%	
	CG (*n* = 16)	0/20	30.71 ± 3.64							
Jung et al. 2022 (57)	CG (*n* = 14)	14/0	22.58 ± 1.69	75.00 ± 0.87	60~80% HRR	12 weeks	3 days/week	1~2 weeks: 25 min	NR	TG, HDL, LDL, TC
	AE (*n* = 14)	14/0	22.50 ± 1.75					3~8 weeks: 40 min		
								9~12 weeks: 55 min		
Salus et al. 2022 (32)	SIT (*n* = 14)	0/14	30.3 ± 3.2	13.40 ± 0.56	82.5 ± 1.5% HR_{max}	12 weeks	3 days/week	SIT: 18 min	NR	TG, HDL, LDL, TC
	CG (*n* = 14)	0/14	32.6 ± 5.9							
Meng et al. 2022 (47)	HIIT (*n* = 15)	0/15	24.5 ± 1.1	11.20 ± 0.20	HIIT: 80% HR_{max}	12 weeks	3 days/week	HIIT: 11 min	NR	TG, HDL, LDL, TC
	MICT (*n* = 15)	0/15	24.4 ± 0.9		MICT: 70% HR_{max}			MICT: 30 min		

	CG (*n* = 15)	0/15	23.8 ± 0.8							
Niu et al. 2023 (51)	BWTCG (*n* = 27)	0/27	26.07 ± 1.30	18.52 ± 0.11	50-70% HR_{max}	12 weeks	3 days/week	30 min	NR	TG, HDL, LDL, TC
	THTCG (*n* = 27)	0/27	26.45 ± 1.12							
	CG (*n* = 27)	0/27	26.23 ± 1.12							
Tan et al. 2016 (35)	AE (*n* = 15)	15/0	28.5 ± 2.1	45–59	62% HR_{max}	10 weeks	5 days/week	60 min	NR	TG, HDL, TC
	CG (*n* = 11)	15/0	27.8 ± 1.5							
Racil et al. 2013 (50)	HIIT (*n* = 11)	11/0	30.8 ± 1.6	15.93 ± 0.60	HIIT: 100%-110% MAS	12 weeks	3 days/week	31-33 min	NR	TG, HDL, LDL, TC
	MIIT (*n* = 11)	11/0			MIIT: 70%-80% MAS					
	CG (*n* = 12)	11/0			(R:50% MAS)					
Nazari et al. 2020 (52)	CG (*n* = 13)	13/0	30.24 ± 2.89	25–35	HIIT: 90-95% HR_{max}	8 weeks	3 days/week	8/10/12/17 min	NR	TG, HDL, LDL, TC

	HIIT (*n* = 14)	14/0	30.92 ± 3.49		MIIT: 75-80% HR_{max}					
	MIIT (*n* = 13)	13/0	31.20 ± 3.37							
Seo et al. 2012 (33)	YOGA (*n* = 10)	0/10	29.04 ± 2.11	14.65 ± 0.28	40~60% HRR	8 weeks	3 days/week	40 min	NR	TG, HDL, LDL, TC
	CG (*n* = 10)	0/10	28.57 ± 1.23							
Saremi et al. 2010 (58)	AE (*n* = 9)	0/9	29.11 ± 1.69	43.1 ± 4.7	80-85% HR_{max}	12 weeks	5 days/week	50-60 min	NR	TG, HDL, LDL, TC
	CG (*n* = 9)	0/9	29.54 ± 1.82							
Mohammadi et al. 2014 (53)	AE (*n* = 15)	0/15	28.67 ± 1.50	27.83 ± 1.69	65-80% HR_{max}	8 weeks	3 days/week	35-55 min	NR	TG, HDL, LDL, TC
	CG (*n* = 15)	0/15	28.49 ± 10.45							
Kim et al. 2007 (49)	AE (*n* = 14)	0/14	29.6 ± 0.6	16.90 ± 0.10	60-90 jump/min	6 weeks	5 days/week	40 min	NR	TG, HDL, LDL, TC
	CG (*n* = 12)	0/12	29.4 ± 0.7							
Keating et al. 2015 (22)	LO: HI (*n* = 12)	7/5	33.9 (0.9)	43.85 ± 2.44	LO: HI, 50% VO_2peak	8 weeks	i) 4 d/week	i) LO: HI, 60 min	LO: HI, 94%;	TG, HDL, LDL, TC
	HI: LO (*n* = 12)	6/6	36.3 (1.7)				ii) 3 d/week	ii) HI: LO, 45 min		

	LO: LO (*n* = 12)	9/3	31.3 (0.8)		HI: LO, 70%		iii) 3 d/week	iii) LO: LO, 45 min	HI: LO,	
	PL (*n* = 12)	9/3	32.2 (1.4)		VO_2peak				90%;	
					LO: LO, 50%				LO: LO,	
					VO_2peak				96%	
Eizadia et al. 2013 (54)	AE (*n* = 17)	0/17	32.1 ± 2.9	43 ± 3	60-80% HR_{max}	12 weeks	3 days/week	45 min	NR	TG
	CG (*n* = 17)	0/17	32.2 ± 2.3							
Chung et al. 2017 (34)	SSG (*n* = 12)	12/0	25.27 ± 3.94	48.21 ± 1.37	70-85% VO_2max	12 weeks	3 days/week	30 min	NR	TG, HDL, LDL, TC
	MSG (*n* = 12)	12/0	24.57 ± 1.76							
	CG (*n* = 12)	12/0	25.39 ± 2.5							
Khammassi et al. 2018 (55)	HIIT (*n* = 6)	0/6	29.3 ± 2.5	18-21	30s: 100% MAV	12 weeks	3 days/week	15-27 min	NR	TG, HDL, LDL, TC
	CG (*n* = 10)	0/10	29.0 ± 2.2		30sR: 50% MAV					
Timmons et al. 2023 (56)	BWHIIT (*n* = 9)	0/9	28.2 ± 1.9	25.70 ± 0.60	Vigorous intensity	8 weeks	3 days/week	30 min	90.8%	TG, HDL, LDL, TC

	CG (*n* = 9)	0/9	27.2 ± 2.3							
Hovsepian et al. 2019 (45)	AE (*n* = 15)	15/0	38.80 ± 2.20	20.45 ± 0.29	85%-90% HR_{max}	10 weeks	4 days/week	40 min	NR	TG, HDL, LDL, TC
	CG (*n* = 15)	15/0	39.90 ± 2.70							
Kim et al. 2014 (46)	CG (*n* = 16)	16/0	26.3 ± 1.3	46.40 ± 0.93	50-60% VO_2max	12 weeks	3 days/week	60-120 min	NR	TG, HDL, TC
	AE (*n* = 16)	16/0	26.0 ± 1.2							

Abbreviations: SD, standard deviation; AE, aerobic exercise; CG, control group; MICT, moderate-intensity continuous training; HIIT, high-intensity interval training; SIT, sprint interval training; MIIT, moderate-intensity interval training; LO: HI, low to moderate intensity, high volume aerobic exercise; HI: LO, high intensity, low volume aerobic exercise; LO: LO, low to moderate intensity, low volume aerobic exercise; PL, placebo; SSG, single-session group; MSG, multiple-session group; THTCG, traditional He-style Tai Chi group; BWTCG, Bafa Wubu Tai Chi group; BWHIIT, bodyweight exercise-based high-intensity interval training; HRR, heart-rate reserve; VO_{2max}, maximal oxygen consumption; VO_2peak, peak oxygen uptake; MAS, maximal aerobic speed; MAV, maximal aerobic velocity; HR_{max}, maximum heart rate; MET, metabolic equivalent; TG, triglycerides; TC, total cholesterol; HDL, high-density lipoprotein cholesterol; LDL, low-density lipoprotein cholesterol; NR, no report.

Table S2. Results of subgroup analysis

Subgroup	Indexes															
	TG				TC				HDL				LDL			
	n	SMD [95%CI]	I^2	*P*	n	SMD [95%CI]	I^2	*P*	n	SMD [95%CI]	I^2	*P*	n	SMD [95%CI]	I^2	*P*
**Interval training**	11	-0.56 [-0.88, -0.24]	40%	0.0007	11	-0.44 [-0.71, -0.17]	19%	0.002	11	0.23 [-0.18, 0.64]	63%	0.27	10	-0.68 [-0.99, -0.37]	25%	< 0.0001
**Continuous training**	16	-0.53 [-0.81, -0.24]	56%	0.0003	15	-0.12 [-0.31, 0.07]	0%	0.21	15	0.40 [0.15, 0.65]	39%	0.002	12	-0.21 [-0.53, 0.10]	53%	0.19
**Moderate-intensity**	12	-0.48 [-0.69, -0.27]	4%	< 0.0001	12	-0.23 [-0.43, -0.02]	0%	0.03	12	0.36 [0.03, 0.68]	56%	0.03	9	-0.49 [-0.76, -0.23]	15%	0.0003
**Vigorous-intensity**	13	-0.66 [-1.06, -0.25]	67%	0.001	12	-0.35 [-0.64, -0.07]	30%	0.01	12	0.25 [-0.10, 0.59]	51%	0.16	11	-0.54 [-0.89, -0.19]	47%	0.003
**Age＜18**	9	-0.41 [-0.73, -0.09]	33%	0.01	9	-0.21 [-0.50, -0.09]	23%	0.17	9	0.04 [-0.32, 0.41]	48%	0.81	7	-0.40 [-0.94, 0.14]	66%	0.15
**18≤Age＜45**	13	-0.69 [-1.06, -0.32]	65%	0.0003	12	-0.27 [-0.53, -0.01]	26%	0.04	12	0.39 [0.06, 0.71]	53%	0.02	12	-0.38 [-0.68, -0.09]	44%	0.01
**45≤Age＜60**	4	-0.39 [-0.78, -0.00]	0%	0.05	4	-0.19 [-0.57, 0.20]	0%	0.34	4	0.68 [0.23, 1.13]	20%	0.003	2	-0.67 [-1.54, 0.20]	54%	0.13
**Overweight (25≤BMI＜30)**	16	-0.49 [-0.68, -0.30]	0%	< 0.00001	16	-0.22 [-0.41, -0.03]	0%	0.02	16	0.46 [0.20, 0.71]	41%	0.0004	13	-0.47 [-0.77, -0.17]	46%	0.002
**Obesity (BMI≥30)**	11	-0.65 [-1.11, -0.18]	73%	0.007	10	-0.28 [-0.60, 0.04]	40%	0.09	10	0.14 [-0.25, 0.52]	58%	0.49	9	-0.34 [-0.72, 0.05]	52%	0.09

Abbreviations: TG, triglyceride; TC, total cholesterol; HDL, high-density lipoprotein; LDL, low-density lipoprotein; CI, confidence interval.

Table S3. Meta-regression coefficients of the effect of significant moderators on selected outcomes

Index	β (95% CI)						
	Weekly deficits (kcal/week)	Session duration (min)	Frequency (times/week)	Relative intensity (VO_2peak)	Relative intensity (HR_{max})	METs	Duration（weeks）
TG	-0.0003 [-0.0008, 0.0002]	-0.002 [-0.014, 0.01]	0.0530 [-0.141, 0.247]	0.0043 [-0.012, 0.021]	0.006 [-0.017, 0.029]	0.056 [-0.114, 0.225]	0.0002 [-0.123, 0.123]
TC	0.0002 [-0.0001, 0.0006]	0.004 [-0.004, 0.0115]	0.107 [-0.023, 0.237]	-0.005 [-0.016, 0.006]	-0.007 [-0.022, 0.008]	-0.061 [-0.175, 0.054]	-0.056 [-0.139, 0.027]
HDL	0.0001 [-0.0005, 0.0005]	0.002 [-0.010, 0.011]	0.020 [-0.167, 0.208]	0.006 [-0.009, 0.0216]	0.009 [-0.012, 0.030]	0.069 [-0.088, 0.225]	0.05 [-0.062, 0.166]
LDL	0.0006 [-0.00002, 0.001]	0.019 [0.002, 0.036] *	0.109 [-0.078, 0.296]	-0.004 [-0.022, 0.014]	-0.005 [-0.030, 0.020]	-0.05 [-0.235, 0.136]	-0.165 [-0.262, -0.069] *

Abbreviations: TG, triglyceride; TC, total cholesterol; HDL, high-density lipoprotein; LDL, low-density lipoprotein; CI, confidence interval; VO_2peak, peak oxygen uptake; HRmax, maximal heart rate; METs, metabolic equivalents.

Table S4. Results of Egger's test

<table>
<tr><th>Index</th><th>Std _ EFF</th><th>Coef.</th><th>Std. Err.</th><th>t</th><th>P ＞ |t|</th><th>95% CI</th></tr>
<tr><td>TG</td><td>Slope</td><td>0.152915</td><td>0.7260044</td><td>0.21</td><td>0.835</td><td>-1.342319, 1.648149</td></tr>
<tr><td></td><td>Bias</td><td>-1.712866</td><td>1.84009</td><td>-0.93</td><td>0.361</td><td>-5.502603, 2.076871</td></tr>
<tr><td>TC</td><td>Slope</td><td>0.2863295</td><td>0.5231192</td><td>0.55</td><td>0.589</td><td>-0.7933355, 1.365995</td></tr>
<tr><td></td><td>Bias</td><td>-1.361655</td><td>1.345672</td><td>-1.01</td><td>0.322</td><td>-4.138984, 1.415675</td></tr>
<tr><td>HDL</td><td>Slope</td><td>0.6967342</td><td>0.714143</td><td>0.98</td><td>0.339</td><td>-0.7771845, 2.170653</td></tr>
<tr><td></td><td>Bias</td><td>-
0.6583187</td><td>1.805069</td><td>-0.36</td><td>0.719</td><td>-4.383799, 3.067161</td></tr>
<tr><td>LDL</td><td>Slope</td><td>-
0.7966342</td><td>0.7782612</td><td>-1.02</td><td>0.318</td><td>-2.420059, 0.8267903</td></tr>
<tr><td></td><td>Bias</td><td>0.9325678</td><td>1.946629</td><td>0.48</td><td>0.637</td><td>-3.12803, 4.993165</td></tr>
</table>

Abbreviations: TG, triglyceride; TC, total cholesterol; HDL, high-density lipoprotein; LDL, low-density lipoprotein; Coef., coefficient; Std. Err., standard error; t, t-test statistic; CI, confidence interval.

Figure S1. Meta-regression analyses results

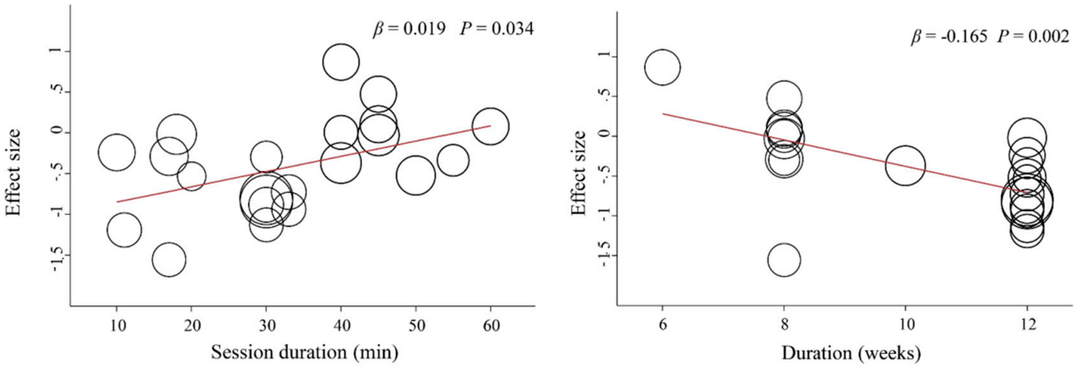

Figure S2. Results of Cochrane risk of bias tool

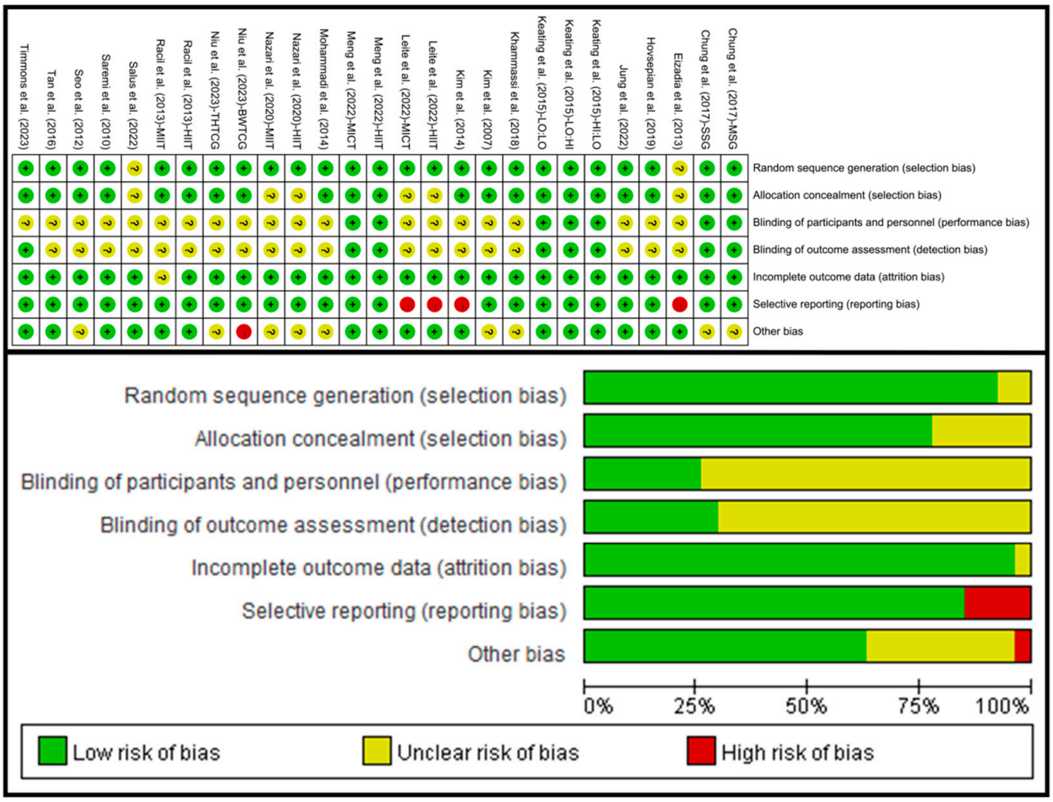

Figure S3. Funnel plot

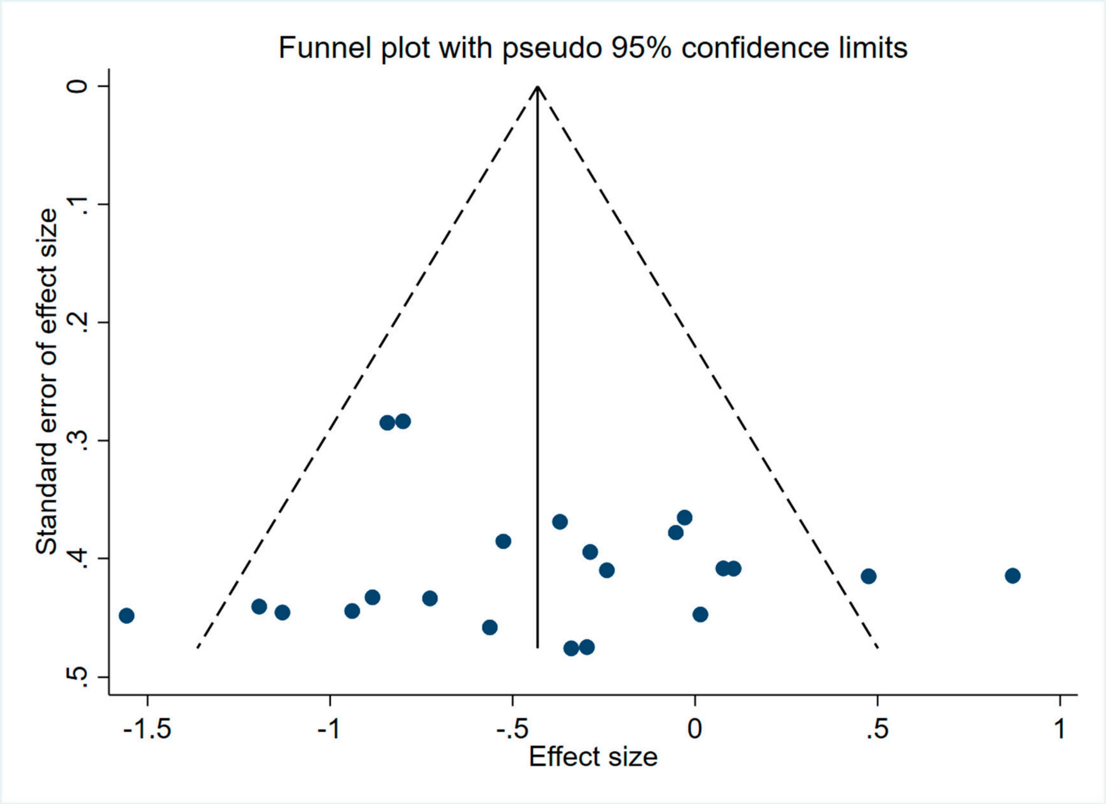

Figure S4. Sensitivity analyses results of TG

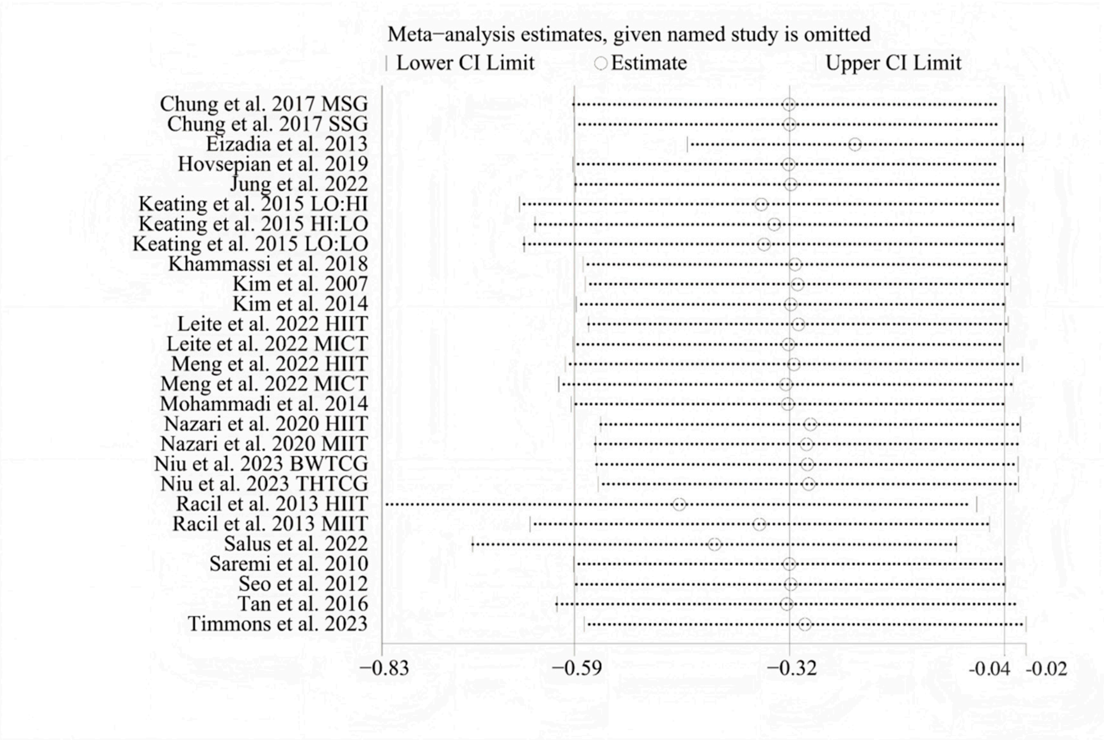

Figure S5. Sensitivity analyses results of TC

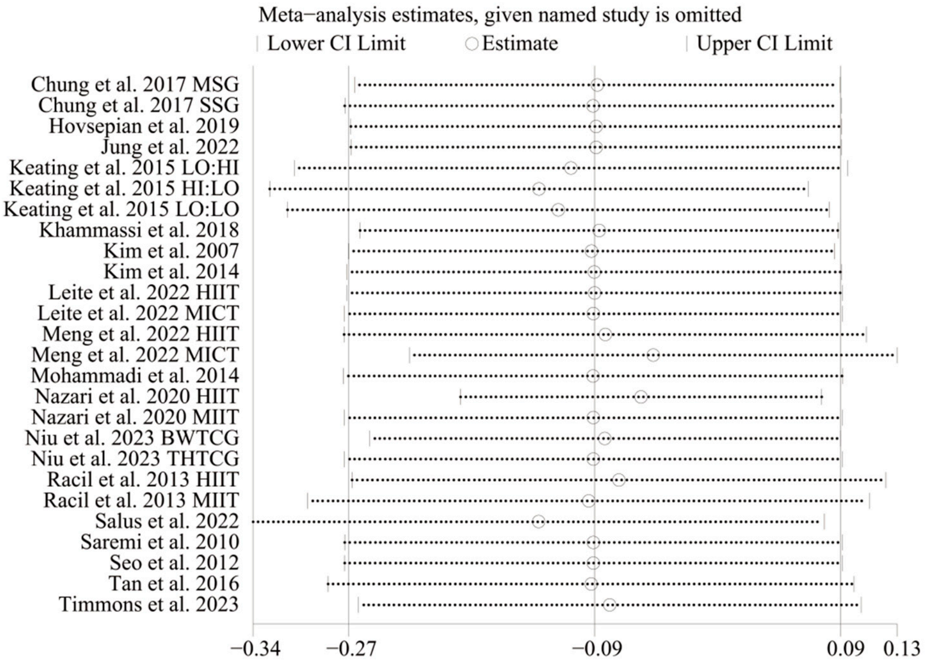

Figure S6. Sensitivity analyses results of HDL

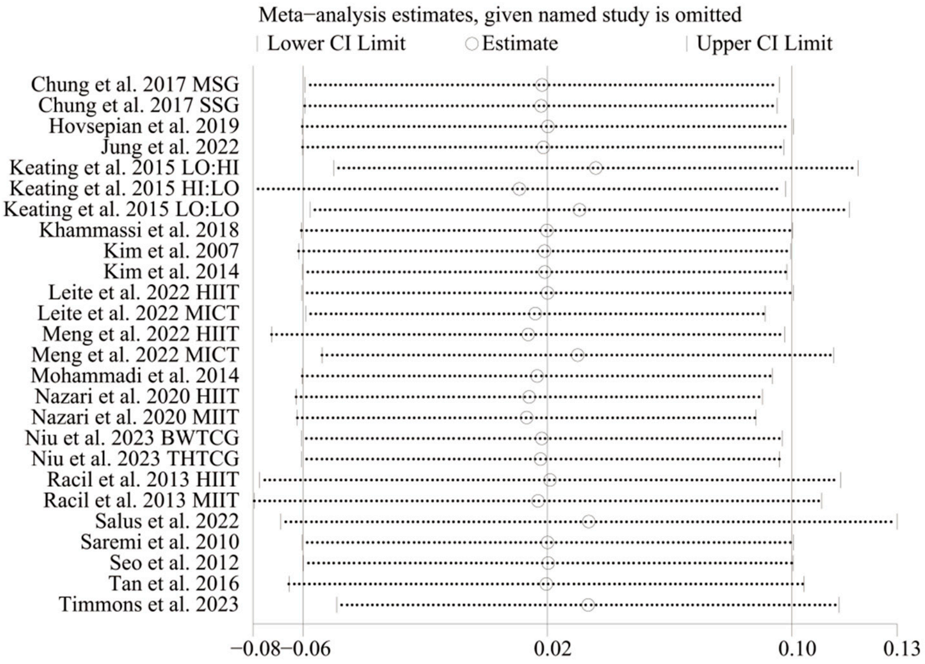

Figure S7. Sensitivity analyses results of LDL

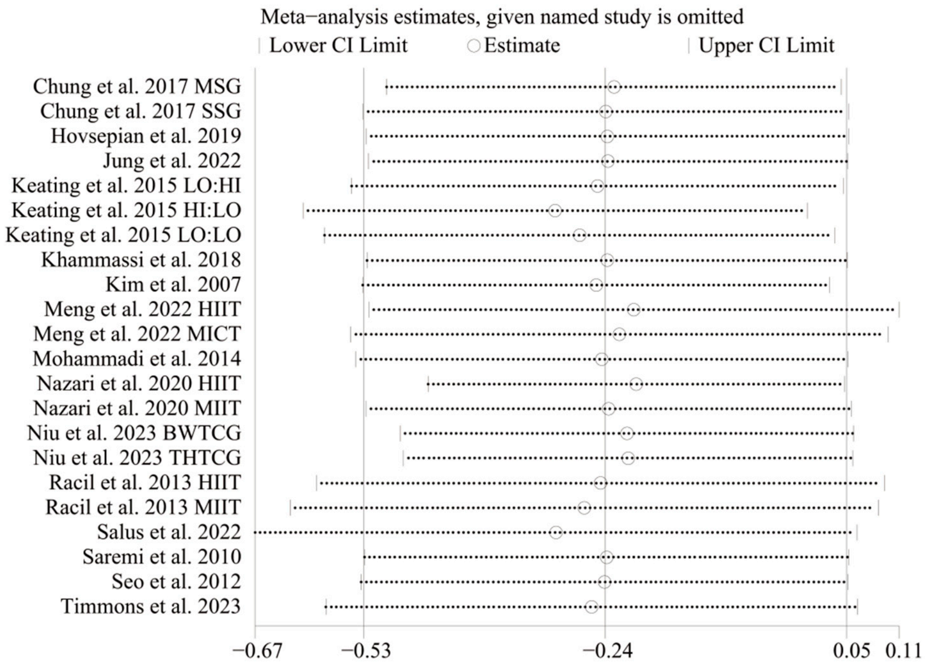

Supplement: Supplementary file 1 [file life-15-00166-s001.zip › life-3420735-supplementary.pdf]
